# Unsuspected Malaria Diagnosed on a Routine Peripheral Smear Review

**DOI:** 10.7759/cureus.61077

**Published:** 2024-05-25

**Authors:** Olanrewaju Oni, Alejandro El Barche, Lekidelu Taddesse-Heath

**Affiliations:** 1 Pathology and Laboratory Medicine, Howard University Hospital, Washington, D.C., USA

**Keywords:** laboratory finding, incidental diagnosis, travel history, plasmodium, malaria diagnosis

## Abstract

Prompt diagnosis of malaria infection is critical for effective management, yet it can be challenging due to varying incubation periods and the need for physician-initiated laboratory workups. We present a case of a 40-year-old male with fever and dark-colored urine, initially evaluated for sepsis. Plasmodium vivax was incidentally identified on a peripheral smear review after obtaining a remote travel history from a malaria-endemic area. Consultation with the Centers for Disease Control confirmed the diagnosis, emphasizing the importance of thorough travel history assessment and timely laboratory investigation in suspected cases of malaria. This case underscores the significance of early diagnosis in managing this potentially life-threatening infection.

## Introduction

Malaria is an infectious disease transmitted to humans by a bite of the female Anopheles mosquito. Malaria infection is prevalent in tropical and subtropical regions throughout the world, with an estimated 247 million cases and 619,000 deaths in 2021, of which 95% of cases and 96% of deaths occurred in Africa [1]. In the United States (US), most malaria cases are seen in people who have traveled to a malaria-endemic region, although few cases of locally acquired malaria infections have been reported [2]. The CDC annually publishes state-level data on malaria cases in the USA, as malaria is a nationally notifiable disease. Over the past few decades, the average number of malaria cases reported to the CDC has steadily increased [3]. In 2017, there were 2,161 confirmed malaria cases in the US, the highest in 45 years, with 86% imported cases from Africa. Around 95% of the patients had symptoms less than 90 days after traveling to a malaria-endemic area [3]. Among individuals returning from overseas, documentation indicates occurrences of all Plasmodium (P.) species, which include P. falciparum, P. vivax, P. malariae, P. ovale, and P. knowlesi; however, P. falciparum and P. vivax make up 69.8% and 9.5%, respectively, of the identified malaria cases. [3].

The incubation period of malaria is variable depending on the Plasmodium species, with the incubation period in most cases ranging from seven to 30 days. Plasmodium vivax can have longer incubation periods lasting six or more months, leading to a delay in diagnosis or a missed diagnosis without awareness and a high index of suspicion. Moreover, Plasmodium vivax and ovale have a dormant stage in the liver and may present 45 days to three years after the initial illness [4]. Malaria is associated with various life-threatening complications, such as severe anemia, liver or renal impairment, cerebral malaria, and acute respiratory distress syndrome. [5].

While laboratory tests to identify malaria are available, the tests are performed by the laboratory when specifically requested by the clinical care team. Therefore, it is imperative to ask about travel history for patients presenting with fever and to keep a high index of suspicion even if the travel history appears to be remote. Incidental malaria diagnosis on routine peripheral smear review is very rare because identification of the organism requires directed time and effort prompted by the attending physician's request to the diagnostic laboratory. This case also underscores the importance of suspecting malaria infection by performing malaria antigen testing and blood smears on any patient who becomes ill while traveling from a malaria-endemic area [5].

## Case presentation

**Figure 1:**
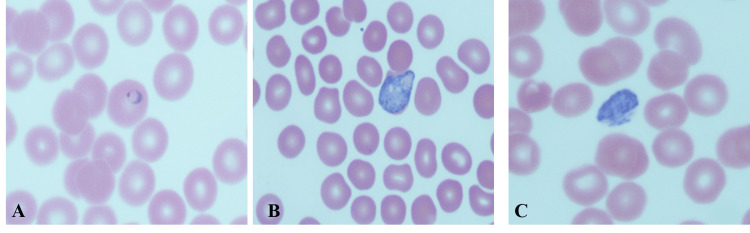
Peripheral smear Plasmodium vivax A: Ring-form trophozoite; B: Developing trophozoite infecting an enlarged RBC with amoeboid features; C: Band-like trophozoites were present mimicking Plasmodium malariae.

A 40-year-old male previously healthy presented with fever (97.3^o^C-101.5^o^C), chills, and dark-colored urine to our emergency department. His blood pressure was 106/65 mmHg; his pulse rate was 88 bpm; and his respiratory rate was 16 cpm. Laboratory workup was initiated with a comprehensive metabolic panel (sodium 134 mEq/L, glucose 116 mg/dL, blood urea nitrogen 20 mg/dL), a complete blood count (platelet 74 x 109, hemoglobin 10.9 g/dL, hematocrit 31.8%), and blood culture. Our automated hematology analyzer, Sysmex XN-2000 (Sysmex America), revealed thrombocytopenia with a normal white blood cell count and hemoglobin; however, the case was flagged by the analyzer for a manual peripheral smear review due to a discrepancy in the white blood cell count between the two different channels that perform the count. A morphologic review of the peripheral smear revealed Plasmodium organisms at different stages, most of which consisted of mature parasite stages with schizonts and gametocytes, and rare ring forms (Figures 1A-1C).

**Figure 2:**
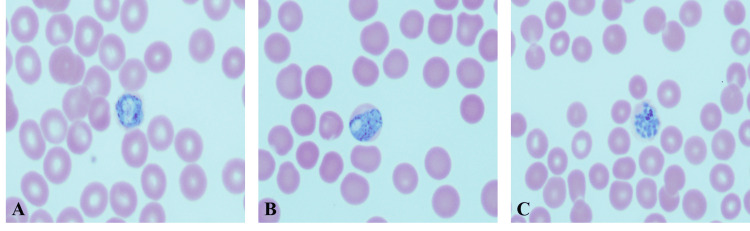
Peripheral smear Plasmodium vivax A, B: Macrogametocytes. The cytoplasm is dark blue with a fine brown pigment. The infected RBCs are large. C: Mature schizonts containing 12-24 merozoites, each with a chromatin dot. There is a clump of pigment, and the infected RBC is enlarged.

Ring forms have a thick cytoplasm with a single chromatin dot. Infected RBCs are larger. Ameboid form and band-like appearance of trophozoite (Figure 1B), enlarged RBC, and Schuffner’s dot; the band form of the trophozoite can also be seen in P. malariae. Gametocytes, the RBC are enlarged. The schizonts are large and amoeboid, with 12-24 merozoites, with each having a dot of chromatin (Figure 2).

**Figure 3:**
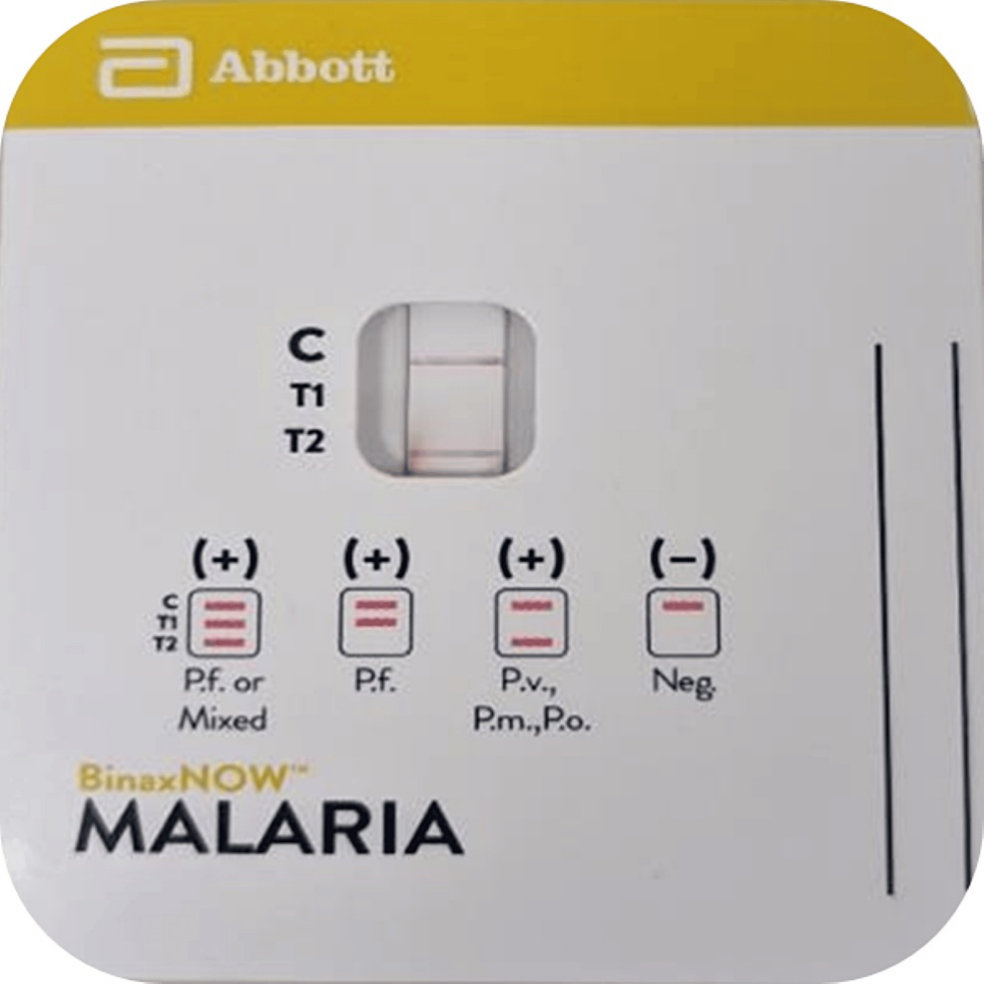
BinaxNOW™ malaria RDT The result was positive for Plasmodium non-falciparum infection (T2 band). C is the control band, the T1 band corresponds to P. falciparum histidine-rich protein 2 (HRP2), and the T2 band corresponds to parasite lactate aldolase. (P. malariae (Pm), P. vivax (Pv), and P. ovale (Po). RDT: rapid diagnostic tests

Based on the morphologic identification, a diagnosis of Plasmodium vivax was made; however, Plasmodium malariae could not be ruled out. A rapid antigen test using the BinaxNOW™ Malaria rapid diagnostic test (RDT) (Abbott Laboratories) was performed, which was positive for Plasmodium, non-falciparum (Figure 3).

The patient's subsequent detailed history revealed that he emigrated to the United States from Venezuela four months prior to his presentation. The patient migration pathway included travel from Venezuela to Colombia and Panama, through the Darien Gap, and finally to Mexico before entering the United States. Due to the prolonged incubation period of four months, consultation was obtained from the Department of Health, District of Columbia, and Center for Disease Control (CDC), Atlanta, Georgia. Based on the peripheral smear images submitted to the CDC, the morphologic diagnosis of Plasmodium vivax to rule out malariae was confirmed. The distinction between the two Plasmodium species was important to rule out the possibility of locally acquired malaria, since a prolonged incubation period is not typical for Plasmodium malariae. Samples were sent to the CDC for PCR confirmation, which was positive for Plasmodium vivax. The patient was treated with antimalarias (atovaquone/proguanil, and later on treated with primaquine), and a reduction in parasitemia was verified by repeated negative peripheral smears. The patient responded positively to the treatment and was eventually discharged for follow-up with a primary care provider.

## Discussion

The timely diagnosis of malaria is essential for effective management and prevention of complications, as the disease can rapidly progress to severe illness, and delayed diagnosis may result in serious complications, including mortality. The decision to conduct a malaria test is typically driven by clinical discretion and the submission of a malaria test order by healthcare providers, who take into account the patient's clinical symptoms and travel history. While the average incubation period for P. vivax is approximately 14 days after exposure, prolonged incubation periods lasting for months to years have been reported [6-9]. In our patient, a malaria test was not ordered since the patient’s travel history exceeded four months. Awareness of the prolonged incubation period is crucial for timely diagnosis. A timely and precise diagnosis of malaria is crucial for preventing disease progression and reducing its severity. The longstanding challenge in epidemiological screening and surveillance lies in achieving accurate diagnosis to detect malaria parasites, which is essential for effective malaria control strategies aimed at reducing morbidity and mortality [10].

Several laboratory tests are available for malaria diagnosis, including rapid diagnostic tests (RDT), microscopic analysis of peripheral blood smears, and polymerase chain reaction (PCR) for definitive species identification. RDTs, which provide results in less than an hour, can detect malaria infections and differentiate Plasmodium falciparum from other species. A study conducted in a US teaching hospital assessed the performance of BinaxNOW™ against microscopy, the gold standard, and found an overall sensitivity of 84.2% and a specificity of 99.8% [11]. Excluding patients on antimalarial therapy, the sensitivity increased to 92.9% [11]. However, BinaxNOW™ initially misidentified a case of P. falciparum as non-falciparum, indicating the need for judicious use in non-endemic areas. Despite the advantages of RDTs, they have limitations, including the risk of false positives and negatives and the inability to measure parasitemia [12]. Therefore, while RDTs are useful for screening, the primary diagnostic test for malaria remains the microscopic examination of peripheral blood smears. Morphological patterns on peripheral smears are used to distinguish different malaria species, though some may overlap and require PCR for accurate detection, which is not widely available and has longer turnaround times. The FDA-approved BinaxNOW™ RDT detects both histidine-rich protein 2 (HRP2) and aldolase antigens, making it valuable for diagnosing malaria. However, its high sensitivity and specificity are mainly for P. falciparum, and its performance may be suboptimal for detecting non-falciparum species like P. vivax [11]. Thus, the gold standard for malaria diagnosis continues to be the microscopic evaluation of peripheral blood smears, which allows for precise identification of Plasmodium parasites.

Nevertheless, this method necessitates time and expertise for accurate interpretation. Incidental diagnosis of malaria on routine review of peripheral smears is very rare since the identification of the organism requires directed time and effort prompted by the attending physicians to the diagnostic laboratory. The utilization of automated hematology analyzers has attracted attention in the preliminary diagnosis of malaria, especially in areas where the disease is endemic [13]. Abnormalities in the scattergram of white blood cells and basophils observed using automated hematology analyzers, have demonstrated utility in the preliminary diagnosis of P. vivax malaria, especially when accompanied by thrombocytopenia, even in cases where malaria is not initially suspected. [13,14]. Moreover, previous studies have investigated the utility of hematology analyzers in rapidly assessing and identifying potential malaria infections through various approaches, such as detecting malaria pigment (hemozoin in monocytes), examining depolarized laser light diffraction patterns, and identifying heightened monocyte activation in screening malaria infection. However, these analyzers rely on additional methods, such as peripheral smears, to confirm the presence of malaria parasites [15,16].

In our case, the initiation of morphological evaluation was prompted by an automated hematology analyzer (Sysmex XN) flag, which indicated a disparity in the white blood cell count observed across two distinct channels responsible for the count on the analyzer. This discrepancy typically arises due to the presence of abnormal cells, or giant platelets, which are recognized as the most frequent causative factors. The predominance of the mature stages of P. vivax, with their larger size, was detected by the instrument as abnormal cells, leading to morphological identification. A retrospective study examining abnormalities detected by the Sysmex XN analyzer in malaria-positive samples revealed a detection rate of 56.2% for abnormalities in Plasmodium species other than falciparum, while only 1.1% of samples with falciparum exhibited abnormalities on the analyzer [16]. Mature parasite stages, such as schizonts or gametocytes, were observed on blood smears among these samples [16]. When associated with thrombocytopenia, a Sysmex XN Plasmodium pattern may serve as a valuable indicator for detecting Plasmodium in unsuspected patients, especially when mature parasite stages are present [16]. This observation is consistent with our findings, where the predominance of mature stages in P. vivax led to the abnormal flag and subsequent identification of the organism through morphologic evaluation of the peripheral smear.

Morphological examination remains the gold standard for malaria diagnosis, yet challenges may arise in distinguishing certain malaria species due to overlapping morphological features. In our case, although P. vivax was suspected, the possibility of P. malariae infection could not be entirely discounted. Additionally, the presence of a prolonged incubation period greater than 120 days, typically uncommon in P. malariae infections, raised concerns about the potential for locally acquired malaria. This concern arises, particularly in light of recent cases of locally acquired mosquito-transmitted Plasmodium vivax malaria in the United States [17]. Subsequent PCR studies conducted at the Centers for Disease Control and Prevention (CDC) confirmed the diagnosis of P. vivax, consistent with an imported infection. While the incidental detection of malaria organisms during routine peripheral smear evaluation facilitated prompt and accurate diagnosis, our case underscores the importance of obtaining a comprehensive travel history and requesting malaria testing to prevent delays or missed diagnoses.

## Conclusions

Timely and accurate diagnosis of malaria is crucial for effective management and prevention of complications, as delays can lead to serious outcomes, including mortality. Automated hematology analyzers have emerged as promising tools for preliminary diagnosis, with anomalies observed in white blood cell distribution scattergrams providing valuable indicators for further investigation. However, morphological examination remains essential, as challenges may arise in distinguishing between malaria species. This case highlights the importance of comprehensive patient evaluation, including travel history and malaria testing, to ensure prompt and accurate diagnosis and prevent delays or missed diagnoses.

## References

[1] (2024). World Malaria Report 2023. https://cdn.who.int/media/docs/default-source/malaria/world-malaria-reports/world-malaria-report-2023-spreadview.pdf?sfvrsn=bb24c9f0_4#:~:text=Suggested%20citation.,%2DNC%2DSA%203.0%20IGO..

[2] (2024). Locally Acquired Cases of Malaria in Florida, Texas, Maryland, and Arkansas. https://www.cdc.gov/malaria/new_info/2023/malaria_US.html.

[3] Mace KE, Lucchi NW, Tan KR (2022). Malaria surveillance - United States, 2018. MMWR Surveill Summ.

[4] Warrell DA, Gilles HM (2002). Essential Malariology, 4Ed.

[5] Chang CY (2023). Clinical characteristics and outcome of severe malaria in Kapit, Sarawak, Malaysian Borneo. J Vector Borne Dis.

[6] Glynn JR, Bradley DJ (1995). Inoculum size, incubation period and severity of malaria. Analysis of data from malaria therapy records. Parasitology.

[7] Brasil P, de Pina Costa A, Pedro RS, da Silveira Bressan C, da Silva S, Tauil PL, Daniel-Ribeiro CT (2011). Unexpectedly long incubation period of Plasmodium vivax malaria, in the absence of chemoprophylaxis, in patients diagnosed outside the transmission area in Brazil. Malar J.

[8] Blackburn D, Drennon M, Broussard K (2023). Outbreak of locally acquired mosquito-transmitted (autochthonous) malaria - Florida and Texas, May-July 2023. MMWR Morb Mortal Wkly Rep.

[9] Cogswell FB (1992). The hypnozoite and relapse in primate malaria. Clin Microbiol Rev.

[10] Fitri LE, Widaningrum T, Endharti AT, Prabowo MH, Winaris N, Nugraha RY (2022). Malaria diagnostic update: from conventional to advanced method. J Clin Lab Anal.

[11] Dimaio MA, Pereira IT, George TI, Banaei N (2012). Performance of BinaxNOW for diagnosis of malaria in a U.S. hospital. J Clin Microbiol.

[12] Calderaro A, Montecchini S, Buttrini M (2021). Malaria diagnosis in non-endemic settings: the European experience in the last 22 years. Microorganisms.

[13] Schwartz E, Parise M, Kozarsky P, Cetron M (2003). Delayed onset of malaria--implications for chemoprophylaxis in travelers. N Engl J Med.

[14] Mubeen KH, Devadoss CW, Rangan RA, Gitanjali M, Prasanna S, Sunitha V (2014). Automated hematology analyzers in diagnosis of Plasmodium vivax malaria: an adjunct to conventional microscopy. Mediterr J Hematol Infect Dis.

[15] Mendelow BV, Lyons C, Nhlangothi P (1999). Automated malaria detection by depolarization of laser light. Br J Haematol.

[16] Pillay E, Khodaiji S, Bezuidenhout BC, Litshie M, Coetzer TL (2019). Evaluation of automated malaria diagnosis using the Sysmex XN-30 analyser in a clinical setting. Malar J.

[17] Dumas C, Bienvenu AL, Girard S, Picot S, Debize G, Durand B (2018). Automated Plasmodium detection by the Sysmex XN hematology analyzer. J Clin Pathol.

